# Neuroligin-3 R451C induces gain-of-function gene expression in astroglia in an astroglia-enriched brain organoid model

**DOI:** 10.1186/s13619-024-00219-5

**Published:** 2025-01-08

**Authors:** Rui Dang, Mridul Dalmia, Ziyuan Ma, Mengmeng Jin, Kushal Aluru, Vincent R. Mirabella, Ava V. Papetti, Li Cai, Peng Jiang

**Affiliations:** 1 Department of Cell Biology and Neuroscience, Rutgers University, 604 Allison Road, Piscataway, NJ 08854 USA; 2 Department of Biomedical Engineering, Rutgers University, 599 Taylor Rd, Piscataway, NJ 08854 USA; 3 Child Health Institute of New Jersey and Department of Neuroscience and Cell Biology, Robert Wood Johnson Medical School, Rutgers University, New Brunswick, NJ 08901 USA

**Keywords:** Human induced pluripotent stem cells, Human embryonic stem cells, Astrogliogenesis, Brain organoid, Neurexin, Neuroligin, Autism, Neuroligin-3 R451C

## Abstract

Astroglia are integral to brain development and the emergence of neurodevelopmental disorders. However, studying the pathophysiology of human astroglia using brain organoid models has been hindered by inefficient astrogliogenesis. In this study, we introduce a robust method for generating astroglia-enriched organoids through BMP4 treatment during the neural differentiation phase of organoid development. Our RNA sequencing analysis reveals that astroglia developed within these organoids exhibit advanced developmental characteristics and enhanced synaptic functions compared to those grown under traditional two-dimensional conditions, particularly highlighted by increased neurexin (NRXN)-neuroligin (NLGN) signaling. Cell adhesion molecules, such as NRXN and NLGN, are essential in regulating interactions between astroglia and neurons. We further discovered that brain organoids derived from human embryonic stem cells (hESCs) harboring the autism-associated NLGN3 R451C mutation exhibit increased astrogliogenesis. Notably, the NLGN3 R451C astroglia demonstrate enhanced branching, indicating a more intricate morphology. Interestingly, our RNA sequencing data suggest that these mutant astroglia significantly upregulate pathways that support neural functions when compared to isogenic wild-type astroglia. Our findings establish a novel astroglia-enriched organoid model, offering a valuable platform for probing the roles of human astroglia in brain development and related disorders.

**Supplementary Information:**

The online version contains supplementary material available at 10.1186/s13619-024-00219-5.

## Background

Astroglia, also known as astrocytes, are a subtype of glial cells in the human brain that are tightly integrated into the central nervous system (CNS). They regulate various CNS functions, including structural support, ion homeostasis, metabolism, neural development, synaptic transmission, maintenance of the blood–brain barrier, and brain damage repair (Ben Haim and Rowitch 2017; Chiareli et al. 2021; Chung et al. 2015; Clarke and Barres 2013; Vasile et al. 2017; Volterra and Meldolesi 2005). Astroglia are also actively involved in many neurological diseases, and pathological changes in astroglia have been identified in both human postmortem tissues and mouse model studies (Clarke and Barres 2013; Kruyer et al. 2023; Ponroy Bally and Murai 2021; Sloan and Barres 2014). Interactions between neurons and astroglia through cell adhesion molecules have been shown to be vital for the development and function of neurons, astroglia, and synapses (Liu et al. 2023; Tan and Eroglu 2021). One such family of cell adhesion molecules is neuroligins (NLGNs) (Südhof 2008). Although the role of NLGNs in neurons has been extensively studied, the function of NLGNs in astrocytes remains unclear. Recent studies in mouse models indicate that interactions between neurons and astrocytes require NLGNs (Dang et al. 2024; Stogsdill et al. 2017). In human pluripotent stem cell (hPSC)-derived brain organoids, NLGN1 enhances astroglial development, highlighting its role in advancing human neural cell maturation (Voss et al. 2023); however, the specific functions of NLGNs in human astroglia have yet to be determined. Additionally, multiple mutations in NLGNs have been identified in patients with autism spectrum disorders (ASD), including the first gene mutation linked to ASDs, namely the NLGN3 arginine-451 to cysteine (R451C) point mutation (Jamain et al. 2003; Trobiani et al. 2020). Studies in NLGN3 R451C knock-in mice and human neurons have demonstrated notable gain-of-function changes, which may contribute to autism-associated phenotypes (Etherton et al. 2011; Tabuchi et al. 2007; Wang et al. 2022).

The pathophysiology of astroglia have been extensively studied using mouse models (Kruyer et al. 2023; Ponroy Bally and Murai 2021). However, substantial differences between humans and mice complicate the investigation of the pathological roles of astroglial in various brain disorders when relying solely on mouse models (Lui et al. 2011; Oberheim et al. 2009, 2006; Zhang et al. 2016). Brain organoids have now emerged as advanced tools for studying human-specific mechanisms of development and disease (Corsini and Knoblich 2022; Okano and Morimoto 2022; Qian et al. 2019; Xu et al. 2021). A current limitation in studying astroglia using organoid models is the inefficient astrogliogenesis. This inefficiency largely stems from the prolonged time required for neural progenitor cells to begin differentiating into glial cells, with astroglia maturation potentially taking up to one year (Sloan et al. 2017; Verkerke et al. 2024). Although considerable efforts have been made to accelerate astrogliogenesis in brain organoids (Voss et al. 2023; Wang et al. 2024) further work is necessary to generate astroglia-enriched organoids that can effectively recapitulate their developmental trajectory in humans. This is especially important for understanding human astroglial biology and the interactions between neurons and astroglia under both physiological and pathological conditions.

In this study, we established a straightforward and effective method to enhance astrogliogenesis in organoids by administering bone morphogenetic protein 4 (BMP4) during the neural differentiation phase. Previous studies have indicated that BMP4 can induce astroglial differentiation in monolayer cultures or at late stages of organoid culture (Gomes et al. 2003; Gross et al. 1996; Jiang et al. 2013; Voss et al. 2023). We observed that the BMP4-treated organoids exhibited robust astrogenesis and complex astroglial structures as early as three weeks post-differentiation. To further assess the improved development of astroglia in our astroglia-enriched organoid compared to time-matched monolayer cultures, we employed a CD44-based purification system to isolate high-purity astroglia from both the monolayer and astroglia-enriched organoid cultures. We then compared the RNA expression profiles of CD44-positive BMP4-induced astroglia from the monolayer with those purified from the astroglia-enriched organoids. This comparison aimed to assess their alignment with human astroglial development and to elucidate potential functional alterations in astroglia. As proof of concept, we generated astroglia-enriched organoids from human embryonic stem cells (ESCs) carrying the autism-associated NLGN3 R451C mutation. Surprisingly, the NLGN3 R451C mutation not only promoted astrogliogenesis but also enhanced the complexity of astrocytes within these organoids. Taken together, we have established an advanced astroglia-enriched organoid model that can be utilized to investigate human astroglial development and the associated pathological changes in neurodevelopmental disorders.

## Results

### Increased astrogliogenesis in astroglia-enriched organoids

**Fig. 1:**
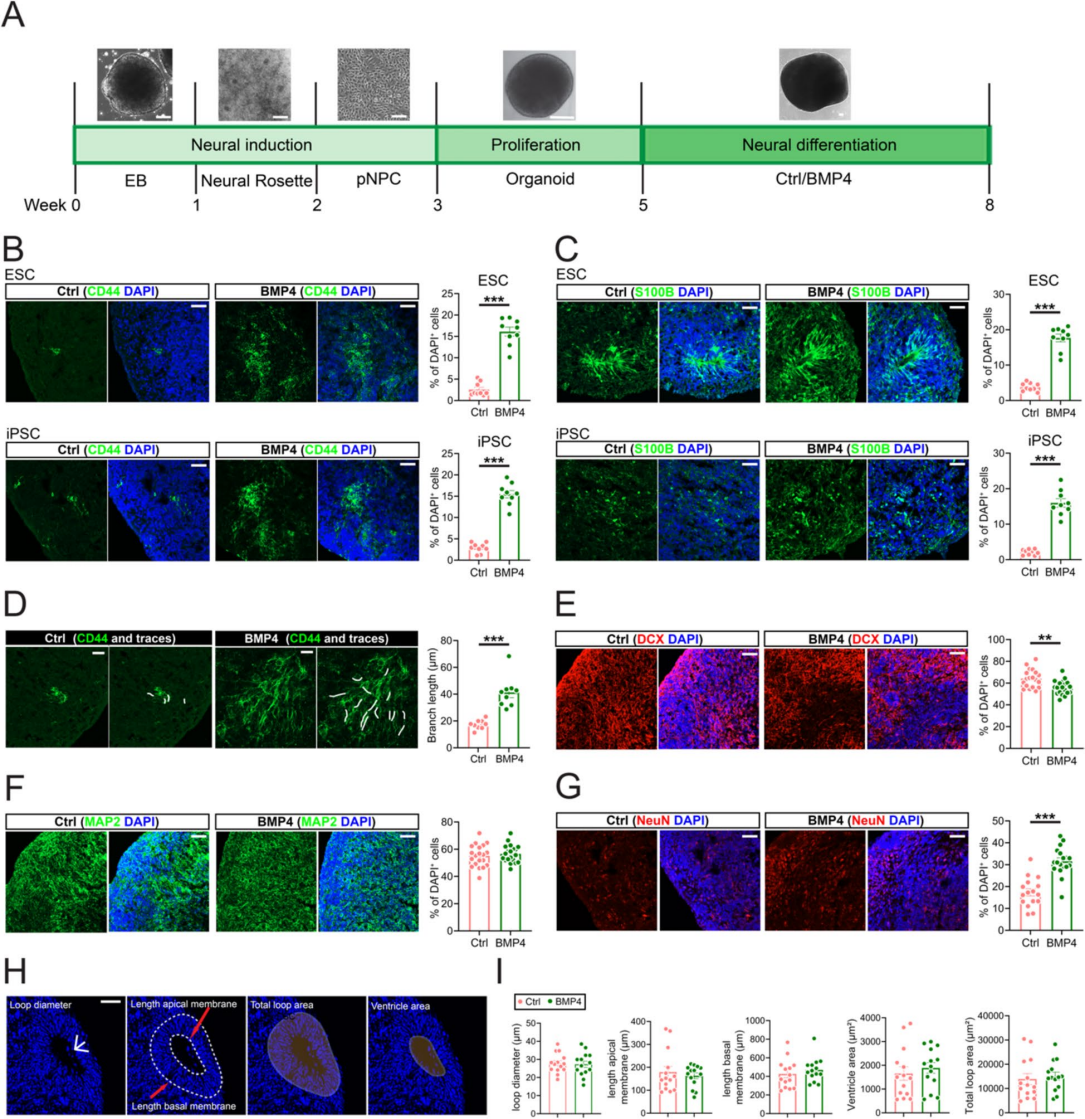
Generation of astroglia-enriched organoids. **A** Illustration of differentiation procedure for deriving astroglia-enriched brain organoids. Scale bar, 100 μm. **B** CD44 staining and quantification of control and astroglia-enriched organoids (*n* = 9 organoids from 3 batches for both ESC and iPSC). Scale bar, 50 μm. **C** S100B staining and quantification of control and astroglia-enriched organoids (*n* = 9 organoids from 3 batches for both ESC and iPSC). Scale bar, 50 μm. **D** Representative images and traces (white lines) and quantification of branch length of CD44^+^ astroglia in control and astroglia-enriched organoids (*n* = 9 organoids from 3 batches of PSC). Scale bar, 20 μm. **E** DCX staining and quantification of control and astroglia-enriched organoids (*n* = 19 organoids from 6 batches of PSC). Scale bar, 50 μm. **F** MAP2 staining and quantification of control and astroglia-enriched organoids (*n* = 20 organoids from 6 batches of PSC). Scale bar, 50 μm. **G** NeuN staining and quantification of control (*n* = 16 organoids from 6 batches of PSC) and astroglia-enriched organoids (*n* = 18 organoids from 6 batches of PSC). Scale bar, 50 μm. **H** Representative images for neural rosette parameter analysis. Scale bar, 50 μm. **I **Quantification of Loop diameter, length apical membrane, length basal membrane, total loop area, and ventricle area of control and astroglia-enriched organoids (*n* = 14 organoids from 4 batches). Data are presented as mean ± SEM. Two-tailed t-test for all panels. **P < 0.01, ***P < 0.001

Brain organoids have emerged as valuable tools for modeling the brain environment essential for astroglia development (Qian et al. 2019; Tan and Eroglu 2021). However, as in human brain development, astrogliogenesis occurs later than neurogenesis in brain organoids. The maturation of astroglia can take over a year, presenting significant obstacles to studying astroglia development and functions during developmental periods in vitro (Qian et al. 2020; Sloan et al. 2017; Verkerke et al. 2024). To enhance astrogliogenesis in brain organoid models, we hypothesized that treatment with the gliogenic molecule bone morphogenetic protein 4 (BMP4) at an early differentiation stage would promote astrogliogenesis in organoids, based on previous studies (Gomes et al. 2003; Gross et al. 1996; Jiang et al. 2013; Voss et al. 2023). To test this hypothesis, we generated organoids from SOX2 and PAX6 double-positive primitive neural progenitor cells (pNPCs; Fig.S1 A, B) according to our previously published protocol (Jiang et al. 2013). To generate astroglia-enriched organoids, we added BMP4 at a concentration of 10 ng/ml—consistent with the concentration used in monolayer astroglia differentiation—following two weeks of proliferation in neural progenitor cell medium (Jiang et al. 2013) (Fig. 1A). Three weeks later, both human embryonic stem cell (hESC) and human induced pluripotent stem cell (hiPSC)-derived organoids exhibited significantly increased expression levels of the astroglia markers CD44 and S100B (Fig. 1B, C). We observed extensive CD44-positive astroglia branches in the BMP4-treated group (Fig. 1D). In addition to astrogliogenesis, the organoids also displayed robust neurogenesis and the formation of neural rosettes (Fig. S1C, D). To assess the effects on neurons, we performed immunostaining experiments using neuronal markers. We conducted immunostaining using neuronal markers, including doublecortin (DCX) for immature neurons, NeuN for mature neurons, and MAP2 as a general neuronal marker. Although the population of MAP2-positive neurons remained unchanged, the astroglia-enriched organoids showed a decrease in DCX-positive neurons and an increase in NeuN-positive neurons, indicating that the enriched astroglia enhanced neuronal maturation in these organoids (Fig. 1E-G). Neural rosette parameters, such as loop diameter, length of apical/basal membrane, and ventricle/total loop area, remain unchanged under BMP4 treatment (Fig. 1H, I). Compared to previous studies, our methods for generating astroglia-enriched organoids demonstrate significant advancements. Notably, our protocol employs a chemically defined medium supplemented with BMP4, avoiding the use of FBS or the overexpression of gliogenic genes such as SOX10, SOX9, and NFIB. Secondly, our protocol enables the generation of astroglia-enriched organoids within just 5 weeks of culture to achieve over 15% astroglia population, whereas other protocols require 6~8 weeks of culture (Allen et al. 2022; Huang et al. 2022; Wang et al. 2024). Taken together, these data demonstrate that BMP4 treatment at the early differentiation stage significantly enhances astrogliogenesis and astroglia development in both hESC and hiPSC-derived brain organoids.

### Enhanced astroglial development and function in astroglia-enriched organoids

**Fig. 2:**
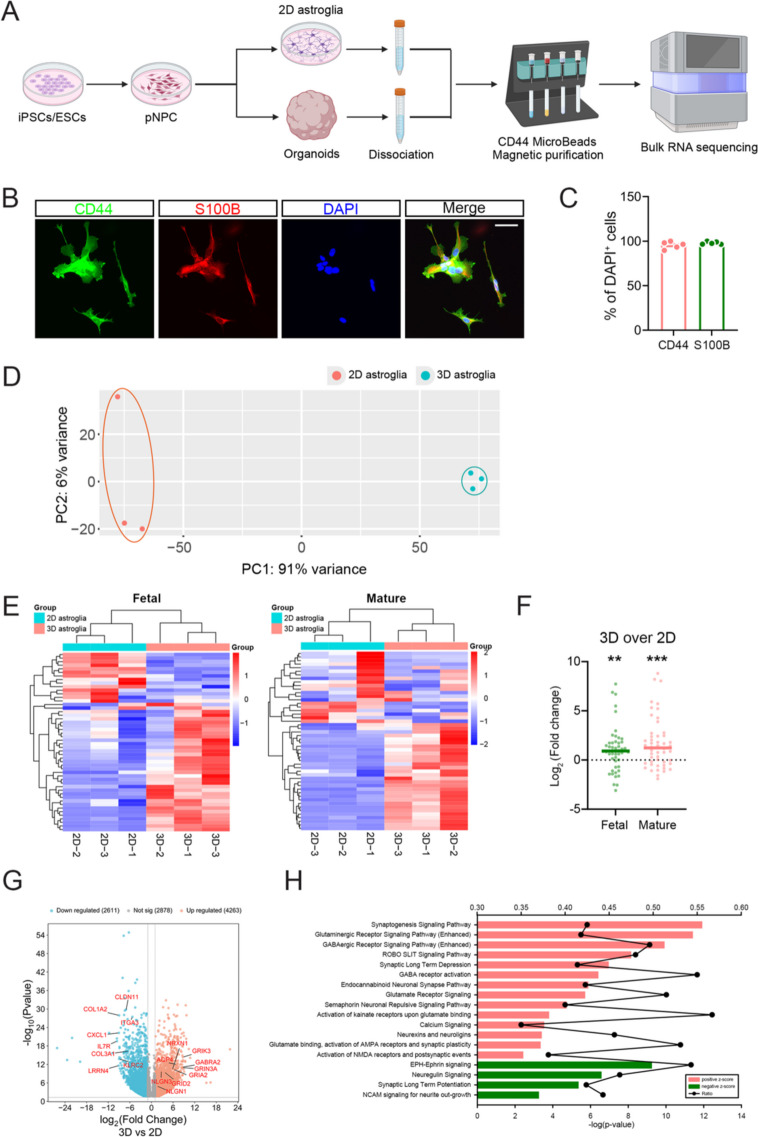
Improved development and enhanced functions of astroglia in astroglia-enriched organoids. **A** Illustration of experimental design. Created by BioRender.com. **B** Representative images of CD44 and S100B staining for cultured purified astroglia. Scale bar, 50 μm. **C** Quantification of the percentage of CD44^+^ and S100B^+^ cells in total DAPI^+^ cells in cultured purified astroglia (*n* = 5 from 3 batches of organoids). **D** PCA plot of 2D and 3D astroglia. **E **Heatmap of top 50 fetal and mature astroglia enriched genes expressed in 2D and 3D astroglia. **F** Quantification of fold change (3D astroglia over 2D astroglia) of top 50 fetal and mature astroglia enriched genes. **G** Volcano plot for upregulated (Red) and downregulated (Blue) DEGs in 3D astroglia versus 2D astroglia. **H** IPA analysis for DEGs in 3D astroglia versus 2D astroglia. Data are presented as mean ± SEM. Two-tailed t-test for all panels. **P < 0.01, ***P < 0.001

Astroglia development requires communication with other neural cell subtypes, such as neurons (Stogsdill et al. 2017). Compared to monolayer cultures of hESC-derived astroglia, astroglia-enriched organoids contain both neurons and neural progenitor cells (Fig. S1C, D). Thus, we hypothesized that our astroglia-enriched organoids would facilitate astroglia development and function compared to time-matched BMP4-induced CD44 and S100B double-positive monolayer astroglia (Fig. S2A, B). To investigate developmental and functional differences between astroglia in our astroglia-enriched organoids (referred to as 3D astroglia) versus monolayer BMP4-induced astroglia (referred to as 2D astroglia), we employed a CD44-based magnetic purification system to isolate CD44-positive astroglia from both 2D and 3D cultures and performed bulk RNA sequencing (Fig. 2A). Additional in vitro culture and staining of the purified 3D astroglia demonstrated a high purity of CD44 and S100B double-positive astroglia post-purification (Fig. 2B, C). After quality control, principal component analysis (PCA) plots showed the 2D and 3D astroglia grouped together, respectively, indicating high data quality and enhancing the reliability of downstream differential expression analyses (Fig. 2D).

To examine potential developmental changes in astroglia, we compared the expression levels of the top 50 human fetal (17~20 gestational weeks) and mature (8~63 years old) astroglia-enriched genes (Table S1) (Zhang et al. 2016). The heatmap revealed that 3D astroglia expressed higher levels of most fetal and mature astroglia genes compared to 2D astroglia, whereas the 2D astroglia only showed increased expression of a few fetal astroglia genes (Fig. 2E). Statistical analysis of the fold change of 3D over 2D astroglia indicated significant upregulation of both fetal and mature astroglia genes (Fig. 2F). These results suggest that 3D astroglia are at a more advanced developmental stage compared to 2D astroglia. Next, to investigate potential functional differences, we identified differentially expressed genes (DEGs) between 3 and 2D astroglia. The volcano plot of DEGs showed comparable numbers of upregulated and downregulated genes in both groups. As pointed out in the volcano plot, among the upregulated DEGs are those related to chemical synaptic transmission and synaptic cell adhesion molecules, which are crucial for synapse development and functions (Fig. 2G). For example, 3D astroglia expressed high levels of synaptic adhesion molecules NLGN1 and NLGN3, confirming the increased synaptic cell adhesion signaling. We also analyzed potential functional changes between 3 and 2D astroglia using IPA analysis. Compared to 2D astroglia, 3D astroglia exhibited enrichment in several pathways related to increased synaptic cell adhesion signaling, synaptic transmission, synaptogenesis, and astroglia activity (Fig. 2H). Notably, the NRXN-NLGN pathway was found to be enriched in the astroglia developed in our organoid model. These observations align with previous studies indicating that the NRXN-NLGN signaling pathway is essential for neuron-astroglia communication and vital for synapse and astroglia development and function (Dang et al. 2024; Stogsdill et al. 2017). Therefore, our astroglia-enriched brain organoid model is not only suitable for investigating astroglia development but also for investigating synaptic development, synaptic transmission, and NRXN-NLGN signaling between neurons and astroglia. In summary, our data suggest that BMP4-induced astroglia-enriched organoids exhibit advanced astroglia development and improved synapse-related functions, including enhanced NRXN-NLGN signaling compared to time-matched monolayer BMP4-induced astroglia.

### Astroglial pathology in NLGN3 R451C mutant organoids

**Fig. 3:**
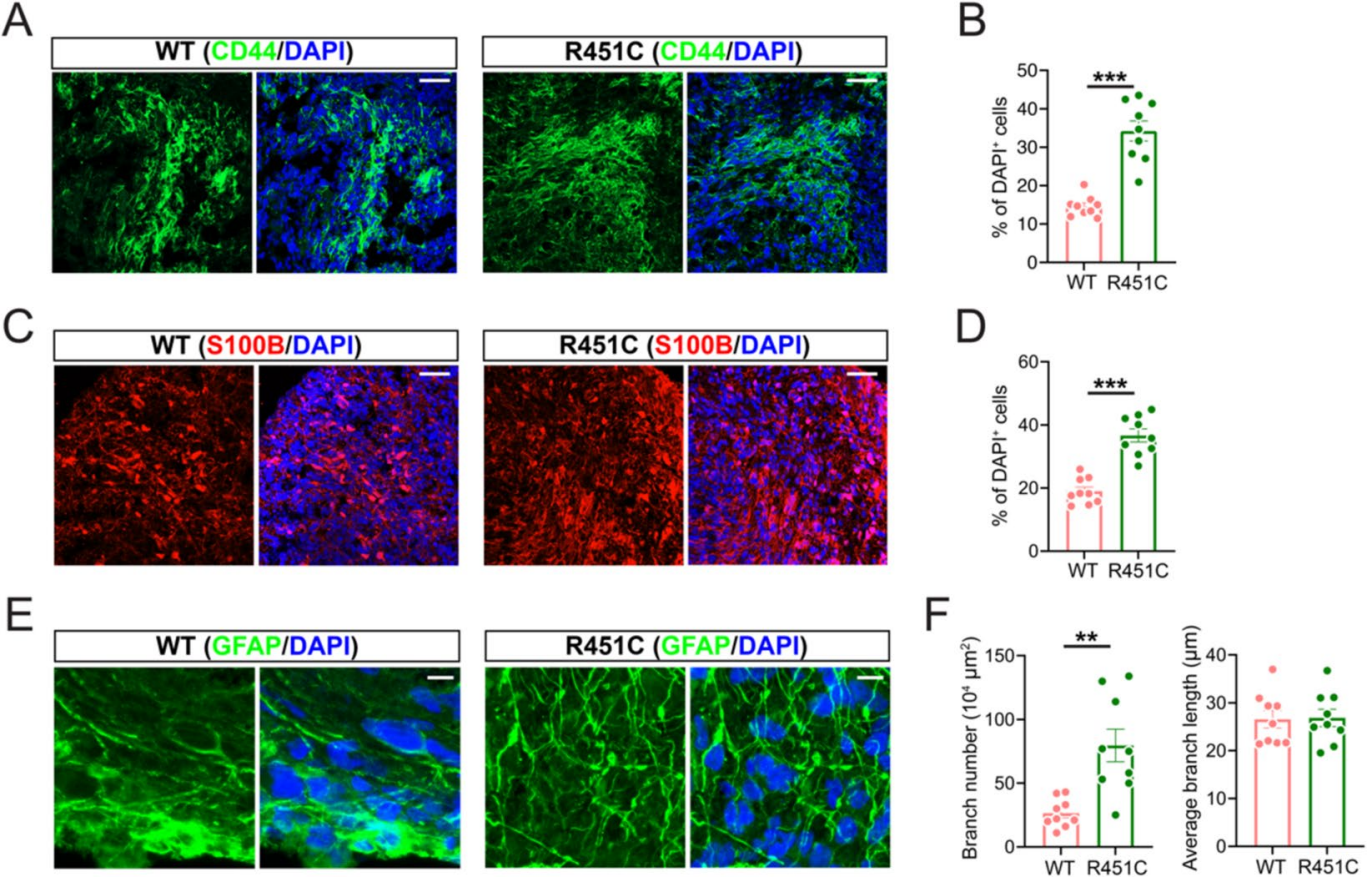
Increased astroglia population and branches in NLGN3 R451C organoids. **A** Representative images of CD44 staining of WT and NLGN3 R451C organoids. Scale bar, 50 μm. **B** Quantification of percentage of CD44^+^ cells in total DAPI^+^ cells in WT and NLGN3 R451C organoids (*n* = 9 organoids from 3 batches). **C** Representative images of S100B staining of WT and NLGN3 R451C organoids. Scale bar, 50 μm. **D** Quantification of percentage of S100B^+^ cells in total DAPI^+^ cells in WT and NLGN3 R451C organoids (*n* = 9 organoids from 3 batches). **E** Representative images of GFAP staining of WT and NLGN3 R451C organoids. Scale bar, 10 μm. **F** Quantification of GFAP^+^ branch number and average branch length in WT and NLGN3 R451C organoids (*n* = 9 organoids from 3 batches). Data are presented as mean ± SEM. Two-tailed t-test for all panels. **P < 0.01, ***P < 0.001

Our astroglia-enriched organoid model provides a valuable opportunity to study NRXN-NLGN signaling, a critical pathway involved in neural development (Dang et al. 2024 Sakers and Eroglu 2019; Südhof 2008). To further investigate astrocytic functions in this pathway, we introduced an autism-associated NLGN3 R451C mutant embryonic stem cell (ESC) line to generate astroglia-enriched organoids. After 35 to 60 days of neural differentiation, we harvested organoids from both the isogenic control (WT) and the R451C mutant (KI) cell lines and performed immunostaining for astrocyte markers. Our CD44 and S100B staining data revealed that R451C mutant organoids exhibited an increased astroglia population compared to WT organoids (Fig. 3A-D). Astroglia extend numerous fine processes that interact with synapses, and their complex structures are critical for synaptic function (Allen and Eroglu 2017; Chung et al. 2015; Oberheim et al. 2006). To this end, we conducted a morphological analysis using GFAP staining and evaluated astroglia complexity. Surprisingly, the NLGN3 mutant astroglia displayed more complex branching compared to WT astroglia, suggesting mutant astroglia have potential synaptic functional enhancements (Fig. 3E, F). Previous studies on NLGN3 knockdown or knockout astroglia reported that the astroglia population remains stable, but the astroglia have either unchanged or decreased complexity (Dang et al. 2024; Qin et al. 2024; Stogsdill et al. 2017). However, our data from the NLGN3 R451C mutation showed both an increased cell population and complexity. This indicates a gain-of-function phenotype, similar to findings in neurons (Etherton et al. 2011; Tabuchi et al. 2007; Wang et al. 2022). In conclusion, these results suggest that the autism-associated NLGN3 R451C mutation promotes astrogliogenesis and advanced astroglia morphology in astroglia-enriched organoids, indicating a potential gain-of-function phenotype.

### Neural supportive gain-of-function in NLGN3 R451C mutant astroglia

**Fig. 4:**
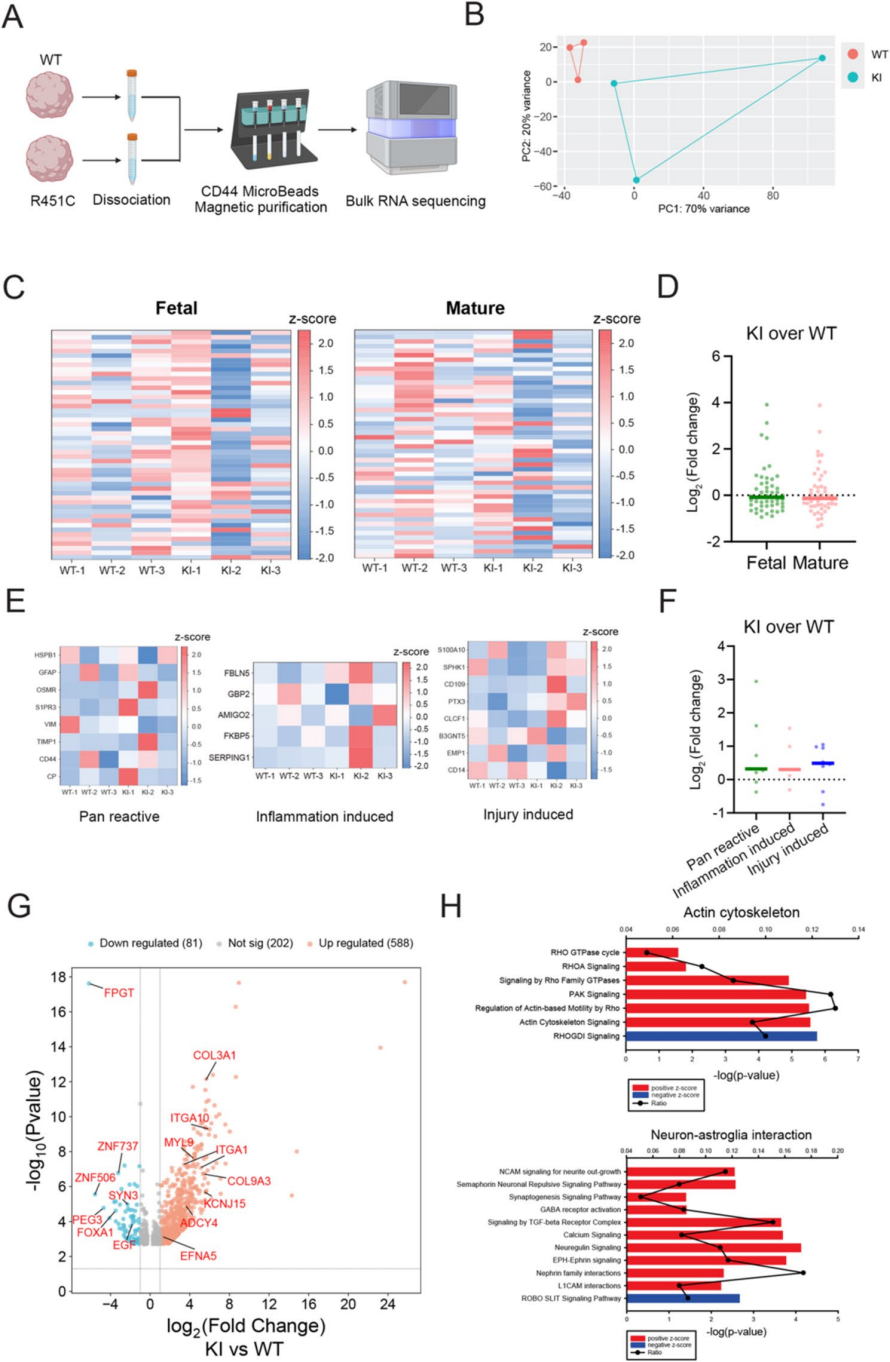
Improved neural functions in NLGN3 R451C astroglia. **A** Illustration of experimental design. Created by BioRender.com. **B** PCA plot of WT and NLGN3 R451C mutant astroglia. **C** Heatmap of expression level of top 50 fetal and mature astroglia enriched genes in WT and NLGN3 R451C mutant astroglia. **D** Quantification of fold change (KI astroglia over WT astroglia) of top 50 fetal and mature astroglia enriched genes in WT and NLGN3 R451C astroglia. **E** Heatmap of the expression level of potential reactive astroglia genes in WT and NLGN3 R451C astroglia. **F** Quantification of fold change (KI astroglia over WT astroglia) of potential reactive astroglia genes in WT and NLGN3 R451C astroglia. **G** Volcano plot for upregulated (Red) and downregulated (Blue) DEGs in NLGN3 R451C astroglia versus WT astroglia. **H** IPA analysis for DEGs in NLGN3 R451C versus WT astroglia. Data are presented as mean ± SEM. Two-tailed t-test for D, one-way ANOVA test with Holm-Sidak test for **F**

We sought to determine whether the morphological changes in KI astroglia contribute to improved synapse development and functions. To address this question, we purified CD44-positive astroglia from both WT and KI organoids and performed bulk RNA sequencing (Fig. 4A). By analyzing the NLGN3 mutation at the mRNA level, we confirmed the presence of the R451C mutation in the purified astroglia (Fig. S3A, B). PCA analysis confirms segregated wildtype and disease astroglia clusters indicating high data quality for downstream differential expression analyses (Fig. 4B). To investigate the potential effects of NLGN3 R451C mutation on astroglia development, we compared the expression levels of the top 50 fetal and mature astrocytic enriched genes (Table S1) (Zhang et al. 2016) between WT and KI astroglia. The data revealed no significant changes in expression level in both fetal and mature astroglia-enriched genes, which indicates developmental timing is maintained in the KI astroglia (Fig. 4C, D). As autism patients exhibit increased astroglia reactivity (Vakilzadeh and Martinez-Cerdeno 2023), we aimed to rule out the possibility of reactive changes in the mutant astroglia by comparing the expression levels of pan-reactive, inflammation-induced, and injury-induced reactive genes (Caldwell et al. 2022) with a threshold of TPM > 2 in our dataset. The results showed that the reactivity state of R451C mutant astroglia remained unchanged compared to WT astroglia (Fig. 4E, F). Next, to assess potential functional changes in mutant astroglia, we conducted DEG analysis between WT and KI astroglia. As pointed out in volcano plot, many genes exhibited increased expression levels in the KI astroglia, particularly genes related to neural development and functions (Fig. 4G). For example, KI astroglia expressed high levels of Ephrin A5 (EFNA5) and Thrombospondin-1 (THBS1), confirming the increased synaptogenesis signaling. Additionally, IPA pathway analysis for R451C mutant astroglia revealed upregulation of pathways related to the actin cytoskeleton and neuron-glia interactions, suggesting enhanced cytoarchitecture and neural supportive functions in the R451C mutant astroglia (Fig. 4H). We observed variations in the NLGN3 R451C KI samples across batches, but the WT samples displayed consistent results. While the PCA plot indicates overall variation in gene expression levels among the samples, our data consistently show that astroglia development and neural supportive genes are expressed at similar levels across all three batches. This consistency strengthens our conclusion that astroglia development remains unchanged and that the neural supportive functions of NLGN3 R451C KI astroglia are improved. Taken together, our data indicate that the NLGN3 R451C mutation in astroglia potentially enhances neural development and functions, consistent with the gain-of-function phenotype observed in neurons.

## Discussion

Human brain organoids represent a sophisticated tool for studying neural development and diseases within the context of human genetics (Corsini and Knoblich 2022; Lancaster et al. 2013). However, investigating astroglia in organoids has been hindered by the delayed onset of astrogliogenesis and the extended maturation periods required to analyze astroglial functions (Sloan et al. 2017; Verkerke et al. 2024). Despite significant efforts to enhance astrogliogenesis in human brain organoids, generating astroglia-enriched organoids remains a considerable challenge (Voss et al. 2023; Wang et al. 2024). BMP4 has been extensively studied for its role in promoting astrogliogenesis in both rodent (Bonaguidi et al. 2005; Gomes et al. 2003; Gross et al. 1996) and human systems (Jiang et al. 2013; Voss et al. 2023). BMP4 promotes astroglial differentiation via SMAD1/STAT3 signaling, where the transcriptional co-activator p300 bridges these pathways, enhancing the expression of astroglia markers such as GFAP (Nakashima et al. 1999). Additionally, BMP4 treatment activates the serine-threonine kinase FKBP12/rapamycin-associated protein (FRAP) also known as the mammalian target of the rapamycin (mTOR) pathway, facilitating STAT3 activation and downstream astroglial differentiation in rodent neural stem cells (Rajan et al. 2003). The cyclin-dependent kinase inhibitor p57kip2 has also been identified as a key upstream regulator of BMP4-mediated astrogliogenesis in rat cultures (Jadasz et al. 2012). However, the capacity for BMP4 to promote astrogliogenesis and development in organoids remains inadequately explored. In this study, we developed a new method to create an astroglia-enriched organoid model specifically for investigating human astroglial development and functions. This new method demonstrates high reproducibility, as evidenced by its effective application across various hESC and hiPSC lines. We incorporated BMP4 into the neural differentiation medium and cultured the organoids for three weeks. This method not only aligns with our 2D astroglial differentiation protocol but also efficiently generates astrocytes within a timeframe comparable to previously reported 2D astrocyte differentiation protocols (Canals et al. 2018; Jiang et al. 2013; Tchieu et al. 2019; Tcw et al. 2017). Our data indicated that BMP4 significantly increased the astroglia population. Notably, the length of CD44-positive branches was also increased significantly compared to the non-treated controls. Give that the morphological complexity of human astrocytes closely correlates with increased functional competence in the adult human brain (Oberheim et al. 2009, 2006; Padmashri et al. 2021), these morphological changes may suggest that our astroglia-enriched organoid model has enhanced functional capabilities, such as neuron-astrocyte communication that is crucial for synapse development and function (Allen and Eroglu 2017; Chung et al. 2015). Indeed, staining data for both immature and mature neuron markers showed a decrease in the immature neuron population and an increase in the mature neuron population in astroglia-enriched organoids, further demonstrating that the enriched astroglia support enhanced neuronal development. Additionally, although beyond the scope of the current study, we propose that a detailed investigation of astroglia-neuron interactions could be undertaken in the future. This could involve ultrastructural analysis using electron microscopy and functional assessments such as glutamate uptake assays and multi-electrode array studies. Moreover, our RNA-seq analysis shows that the 3D astroglia exhibited a significantly more advanced developmental state compared to 2D BMP4-induced astroglia. DEG analysis also revealed the upregulation of genes related to synaptic transmission and synaptic cell adhesion signaling. This includes the upregulation of the NRXN-NLGN signaling pathway, which is highly critical for synapse development and function, as well as astroglia development and function (Dang et al. 2024; Stogsdill et al. 2017). The detailed mechanisms underlying BMP4-induced upregulation of NRXN-NLGN signaling may involve: (1) increased astrogliogenesis, which enhances neuron-astroglia interactions mediated by NRXN-NLGN signaling; and (2) mTOR signaling, a downstream effect of BMP4 treatment. Previous studies have demonstrated that NLGN3 interacts with mTOR signaling in mouse CNS cells (Dang et al. 2024; Venkatesh et al. 2015; Xu et al. 2019a), suggesting that BMP4 may stimulate mTOR signaling and promote NLGN3 activation. Furthermore, the soluble N-terminal fragment of NLGN3 has been shown to activate PI3K-mTOR signaling, which, in turn, increases NLGN3 expression (Venkatesh et al. 2015).

The NRXN-NLGN signaling pathway has been implicated in neurodevelopmental disorders, such as autism and schizophrenia (Chih et al. 2004; Südhof 2008). However, while the role of NLGNs has been widely studied in neurons, the specific role of NLGNs in astroglia remains largely unclear. The NLGN3 R451C mutation is the first autism-associated mutation identified in patients, and studies in mouse and human neurons have shown that this mutation contributes to autism-related phenotypes through a gain-of-function mechanism (Etherton et al. 2011; Tabuchi et al. 2007; Wang et al. 2022). Leveraging our astroglia-enriched organoid models, which exhibit enhanced NRXN-NLGN signaling, we employed hESCs carrying the NLGN3 R451C mutation to develop organoids and further explore astroglial pathology in autism. Our data indicated that the NLGN3 R451C mutation not only drives significant astrogliogenesis in brain organoids but also leads to dramatic morphological changes without affecting the developmental trajectory and reactivity of astroglia. As changes in morphological complexity may reflect functional variations, we analyzed the gene expression levels of CD44-positive NLGN3 R451C mutant astroglia purified from brain organoids. RNA sequencing analysis revealed upregulation of actin-cytoskeleton and neuron-astroglia interaction pathways in NLGN3 R451C astroglia, suggesting an improved role of mutant astroglia in supporting neural functions. Previous mouse studies have shown that the knockdown of NLGN3 during the developmental stage results in decreased astroglia complexity (Stogsdill et al. 2017), while knockout of astroglia NLGN3 in the adult stage leads to decreased synaptic function without affecting astroglia morphology (Dang et al. 2024). These data indicate a loss-of-function phenotype of astroglial NLGN3. However, the NLGN3 R451C mutation in our organoids exhibited increased neural supportive functions, indicating a gain-of-function phenotype, which is consistent with findings in both mouse and human studies of NLGN3 R451C mutation (Etherton et al. 2011; Tabuchi et al. 2007; Wang et al. 2022).

## Conclusions

In conclusion, we established an astroglia-enriched brain organoid model that provides a valuable platform for unraveling astroglia development and pathologies within a human context. Our results indicate that BMP4-treated astroglia cultured in 3D brain organoids produce astroglia with robust morphological and functional enhancements. Notably, astroglia derived from NLGN3 R451C mutant models exhibited a clear gain-of-function alternation. However, our current study is limited to examining the NLGN3 R451C mutant astroglia in isolation. Future investigations into the interactions between NLGN3 R451C astroglia and neurons harboring the same mutation in our new model will be particularly intriguing. Despite these limitations, the enhanced role of autism-related NLGN3 R451C mutant astroglia suggests a significant pathological contribution of astroglia to the development of autism spectrum disorder (ASD), indicating potential targets for future ASD treatments.

## Materials and methods

### Human iPSC lines

A total of one human induced pluripotent stem cell (hiPSC) line and two human embryonic stem cell (hESC) lines were utilized in this study. The hESC lines comprised one healthy control line (hESC H1) and one isogenic NLGN3 R451C knock-in line. All cell lines were fully characterized in previous studies (Jin et al. 2022; Wang et al. 2022). For the NLGN3 R451C knock-in cell line, DNA was extracted and amplified using PCR, with a MluI restriction enzyme site employed for screening the PCR products. The R451C mutation was further validated through RNA mutation analysis in the RNA sequencing data. The two hESC lines (hESC H1 and NLGN3 R451C) were kindly provided by Dr. Zhiping Pang from Rutgers Robert Wood Johnson Medical School.

### Human ESC and iPSC lines culture

All human ESC and iPSC lines were fully characterized in previous studies. The ESC and iPSC lines were cultured on hESC-qualified Matrigel (35 Corning, product #: 354277 ) coated 6-well plates in mTeSR Plus media (STEMCELL Technologies, 100–0276). Passaging of the ESC and iPSC lines occurred every 5~7 days using ReLeSR media (STEMCELL Technologies, 100–0484) with CEPT (50 nM Chroman 1 (MedChem Express, HY-15392), 5 μM Emricasan (Selleckchem, S7775), Polyamine supplement (Millipore, P8483), 0.7 μM Trans-ISRIB (Tocris, 5284)) added to the medium on the first day (Chen et al. 2021).

### Differentiation and culture of pNPCs

pNPCs were generated using our previously published protocol (Xu et al. 2019b). Briefly, ESCs and iPSCs were detached to form neural embryoid bodies in mTeSR Plus media, followed by a 7-day culture in EB medium (DMEM/F12 (Hyclone, SH3002201), 1 × N2 (Thermo Fisher Scientific, 17502,048), SB431542 (10 μM, Tocris, 1614), and noggin (40 ng/ml, Peprotech, 120-10C-50UG)). EBs were then cultured on growth factor-reduced Matrigel (Corning, 354,230)-coated plates in a medium consisting of DMEM/F12, 1 × N2, and laminin (1 μg/ml; Corning, 354,232) to form neural rosettes. Neural rosettes were manually isolated and cultured in pNPC medium, which is a 1:1 mixture of Neurobasal (Thermo Fisher Scientific, 21,103–049) with 1 × GlutaMAX (Thermo Fisher Scientific, 35050061) and DMEM/F12, supplemented with 1 × N2, 1 × B27-RA (Thermo Fisher Scientific, 12587010), FGF2 (20 ng/ml, Peprotech, 100-18B), human leukemia inhibitory factor (hLIF, 10 ng/ml, Millipore, LIF1005), and CHIR99021 (3 μM, Biogems, 2520691), along with SB431542 (2 μM). pNPCs were passaged and expanded using TrypLE Express (Thermo Fisher Scientific, 12605028), with CEPT added to the medium on the first day of all passaged cells. Organoids were generated from pNPCs within 6 passages.

### In vitro differentiation of pNPCs to astroglia

Astroglia were differentiated with minor modifications of our previously published protocols (Chen et al. 2014; Jiang et al. 2013). Briefly, to differentiate pNPCs into astroglia, pNPCs were cultured in suspension for 7 days in NPC medium, which consisted of a 1:1 mixture of Neurobasal with 1 × GlutaMAX and DMEM/F12, supplemented with 1 × N2, 1 × B27-RA, and FGF2 (20 ng/ml). After this period, the neurospheres were dissociated into single cells and plated on growth factor-reduced Matrigel-coated plates in NPC medium for the first two days. The medium was then changed to astroglia differentiation medium containing DMEM/F12, 1 × N2, 1 × B27-RA, BMP4 (10 ng/ml; Peprotech, 120-05ET), and FGF2 (20 ng/ml). The medium was replaced every other day. Astroglia cultured for 20 to 40 days were used in this study.

### Brain organoid culture

Brain organoids were generated using ultra-low-attachment 96-well plates, seeding 10,000 pNPCs in NPC medium supplemented with CEPT for three days. After this initial phase, the organoids were transferred to ultra-low-attachment 6-well plates and cultured in NPC medium on an orbital shaker at a speed of 80 rpm until day 14. On day 14, the medium was changed to a neural differentiation medium consisting of a 1:1 mixture of Neurobasal with 1 × GlutaMAX and DMEM/F12, supplemented with 1 × N2, 1 × B27 (Thermo Fisher Scientific), BDNF (10 ng/ml, Peprotech, 450–02), GDNF (10 ng/ml, Peprotech, 450–10), dibutyryl-cyclic AMP (1 μM, Sigma, D0260), and ascorbic acid (200 nM, Sigma, A4403), with or without BMP4 (10 ng/ml). The medium was replaced every other day. The organoids were utilized after three weeks of differentiation.

### Magnetic isolation of human astroglia

Organoids were dissociated using a 1:1 mixture of TrypLE Express and Accutase (Thermo Fisher Scientific, 00–4555-56) for 30 min at 37 °C with 5% CO₂, gently pipetting to assist in the process. DMEM/F12 was then added to stop the digestion, followed by DNase I (Roche, 04536282001) treatment. The cells were filtered through a 0.22 µm filter to obtain a single-cell suspension. To isolate astroglia, a magnetic-based CD44^+^ MicroBeads system was employed according to the manufacturer's protocol (Miltenyi Biotec, 130–095–194). Briefly, cell pellets were resuspended in 80 µL MACS buffer (0.5% BSA + 2 mM EDTA in PBS) and mixed with 20 µL MicroBeads, incubating at 4 °C in the dark for 15 min. The samples were then processed through LS columns using the MidiMACS separator (Miltenyi). Finally, human CD44^+^ cells were washed into 15 mL tubes for RNA extraction or culture. The purity of the isolated human astroglia was verified through CD44 and S100B staining on cell-seeded coverslips.

### Immunostaining, image acquisition

Organoids were washed with PBS and fixed in 4% paraformaldehyde (PFA) at room temperature for 2 h. Following fixation, they were dehydrated in 25% sucrose at 4 °C. The organoids were then immersed in OCT (optimal cutting temperature compound) and frozen for sectioning. Frozen organoids were cryo-sectioned into 20-μm thick slices for immunofluorescence staining. The slices were washed with PBS three times, then permeabilized and blocked with 5% goat serum and 0.2% Triton X-100 for 1 h at room temperature. Primary antibodies were diluted in 5% goat serum and incubated overnight at 4 °C. After washing the slices with PBS three times, secondary antibodies, also diluted in 5% goat serum, were applied and incubated for 1 h at room temperature. Following a final wash with PBS five times, the slides were mounted using anti-fade Fluoromount-G medium containing DAPI (1,40,6-diamidino-2-phenylindole dihydrochloride) (Southern Biotechnology). The primary antibodies used in this study: anti-PAX6 (GeneTex, GTX113241), anti-SOX2 (Santa Cruz, sc-365823), anti-CD44 (Abcam, ab6124), anti-S100B (Millipore, ABN59), anti-GFAP (Millipore, AB5804), anti-MAP2 (Millipore, MAB3418), anti-DCX (CST, 4604S), anti-NeuN (Sigma, ABN78); The secondary antibodies used in this study Goat anti-mouse 488 (Thermo Fisher Scientific, A-11029), Goat anti-mouse 594 (Thermo Fisher Scientific, A-11032), Goat anti-rabbit 488 (Thermo Fisher Scientific, A27034), Goat anti-rabbit 594 (Thermo Fisher Scientific, A-11037): All images were acquired using a Zeiss 800 confocal microscope and a Keyence microscope (BZ-X810). Images were analyzed via Fiji software (NIH).

### RNA isolation and bulk RNA-seq

Total RNA was extracted using RNeasy Micro Kit (QIAGEN). RNA sequencing was performed by Admera Health, LCC. In brief, after QC test, libraries were constructed utilizing a TruSeqV2 kit from Illumina (Illumina, San Diego, CA) following the manufacturer’s protocol. The libraries were subjected to 75 bp paired read sequencing using a NextSeq500 Illumina sequencer to generate approximately 30 to 35 million paired-reads per sample. Fastq files were generated using the Bcl2Fastq software, version 1.8.4. Quality control was performed using FastQC (v0.12.1) and the fastq files were aligned to the human genome (GRCh38) using HISAT2 (v2.2.1). Count matrices were generated using featureCounts (v2.0.5) with parameters for paired-end and reversely stranded reads. Raw gene counts were processed, and differential gene expression analysis was performed using DESeq2 (v1.42.1) in R (v4.3.2). Log-fold change shrinking was performed using the Ashr method (using the relevant parameter in DESeq2), and pairwise DEG analysis was performed using the contrast functionality of DESeq2. Only genes with a significant adjusted *p*-value (*p*-adj < 0.05) were included for further DEG analysis. Gene ontology enrichment and pathway analysis was performed using IPA (QIAGEN, Summer 2024 release), with upregulated and downregulated genes being enriched together. Pathway analysis results show in the paper use terms from IPA enrichment. For heatmap generation, transcripts per million (TPM) normalization was used before converting expression data to the z-scores presented in the heatmaps. NLGN3 R451C mutation rate was analyzed through Integrative Genomics Viewer software.

### Quantification and statistical analysis

All data are presented as mean ± SEM and were statistically evaluated using a two-tailed t-test. A *p*-value of < 0.05 was considered significant (*P < 0.05, **P < 0.01, ***P < 0.001). All analyses were conducted using SigmaPlot 12.5 (Systat) and Graphpad Prism 9 (GraphPad Software). Each experiment was performed independently at least three times with similar results.

## Supplementary Information

Supplementary Material 1. Fig. S1 Characterization of human PSC-derived pNPCs and organoids. Fig. S2 Characterization of human PSC-derived monolayer astroglia.­ Fig. S3 Characterization of NLGN3 R451C mutation in purified astroglia.

Supplementary Material 2: Table S1. Raw data for Fig. 2E and 4C.

## Data Availability

All data are available from the corresponding author upon request. All RNA sequencing data are available at the National Center for Biotechnology Information (NCBI) under accession number GSE283484.

## Abbreviations

CNS: Central nervous system
NLGN: Neuroligin
NRXN: Neurexin
ASD: Autism spectrum disorder
R451C: Arginine-451 to cysteine
BMP4: Bone morphogenetic protein 4
ESC: Embryonic stem cell
pNPCs: Primitive neural progenitor cells
iPSC: Induced pluripotent stem cell
DEGs: Differentially expressed genes
EBs: Embryoid bodies
