# Supplementary material for: Neuroligin-3 R451C induces gain-of-function gene expression in astroglia in an astroglia-enriched brain organoid model
Source: Cell Regen. 2025 Jan 8;14:1. doi: 10.1186/s13619-024-00219-5 (PMC11711438; doi:10.1186/s13619-024-00219-5)
Supplement: Supplementary file 1 — Supplementary Material 1. Fig. S1 Characterization of human PSC-derived pNPCs and organoids. Fig. S2 Characterization of human PSC-derived monolayer astroglia.­ Fig. S3 Characterization of NLGN3 R451C mutation in purified astroglia. [file 13619_2024_219_MOESM1_ESM.docx]

**Supplementary Figures**


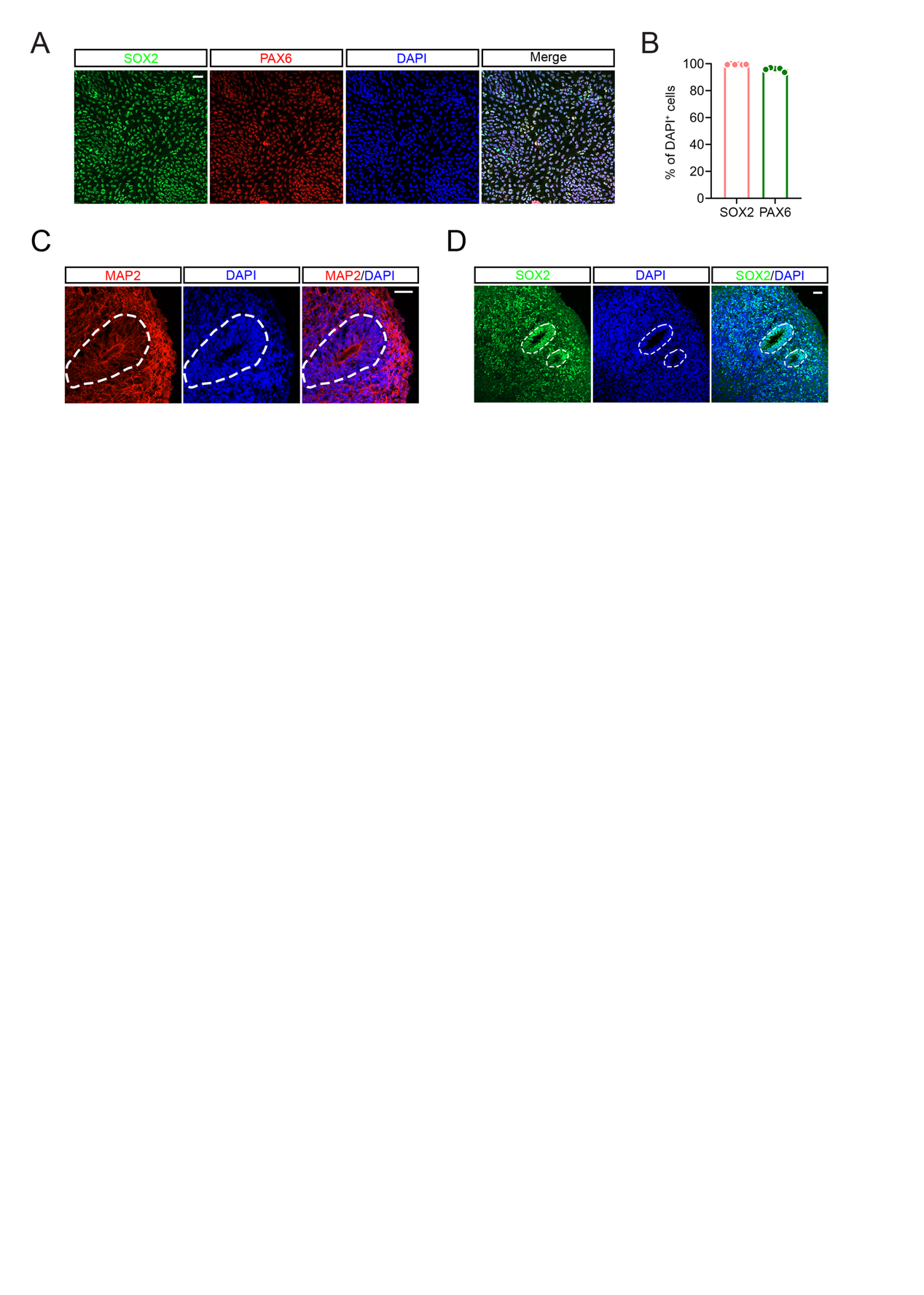


**Fig. S1** Characterization of human PSC-derived pNPCs and organoids.

**A** SOX2 and PAX6 staining of pNPCs. Scale bar, 50 μm.

**B** Quantification of the percentage of SOX2^+^ and PAX6^+^ cells in total DAPI^+^ cells in pNPCs (n = 6 from 3 independent experiments).

**C** MAP2 staining of glia-enriched organoids. Scale bar, 50 μm.

**D** SOX2 staining of glia-enriched organoids. Scale bar, 50 μm.


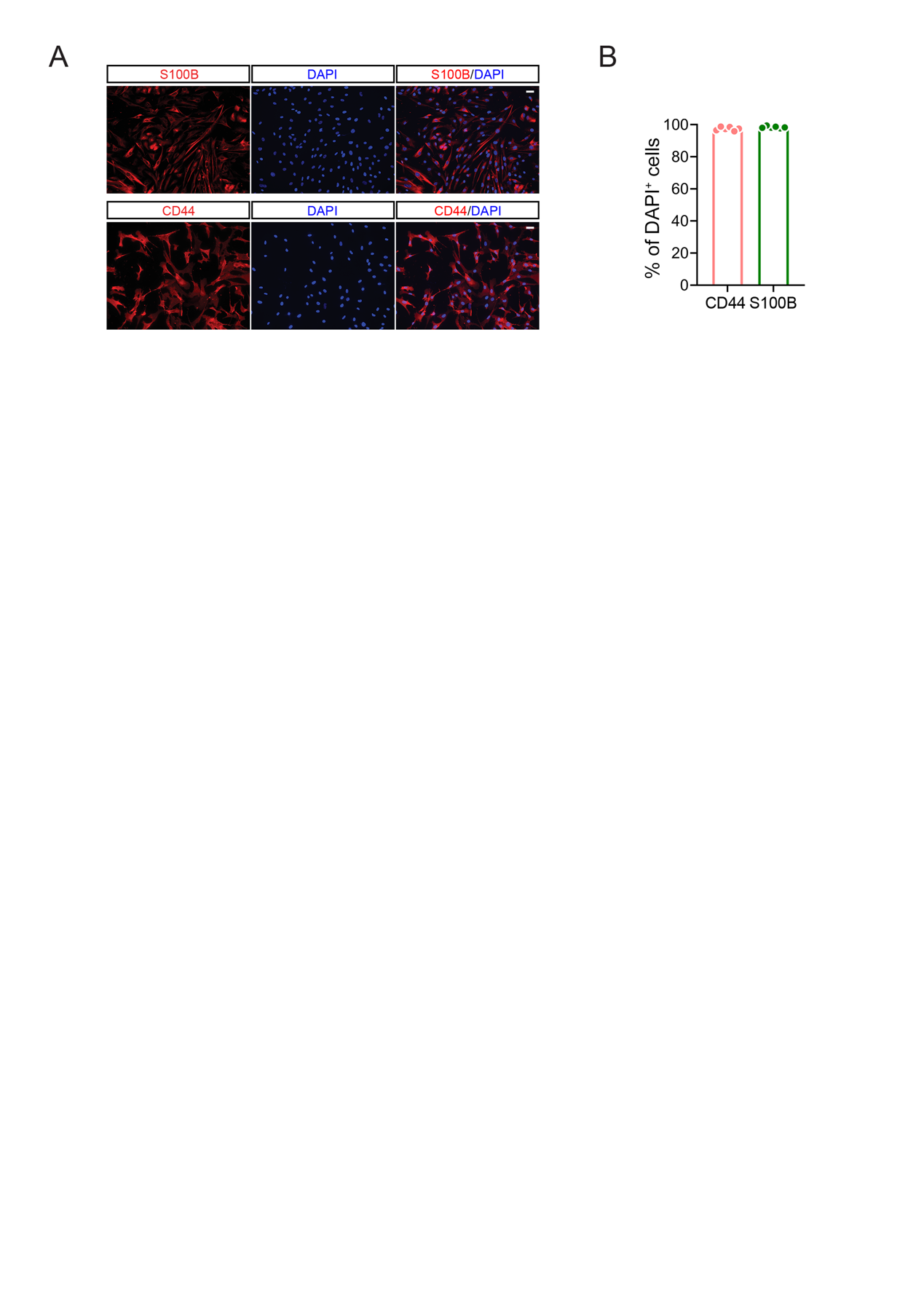


**Fig. S2** Characterization of human PSC-derived monolayer astroglia.­

**A** Representative images of S100B and CD44 staining of 2D cultured astroglia. Scale bar, 20 μm.

**B** Quantification of percentage of CD44^+^ and S100B^+^ cells in total DAPI^+^ cells in 2D cultured astroglia (n = 6, from 3 independent experiments).


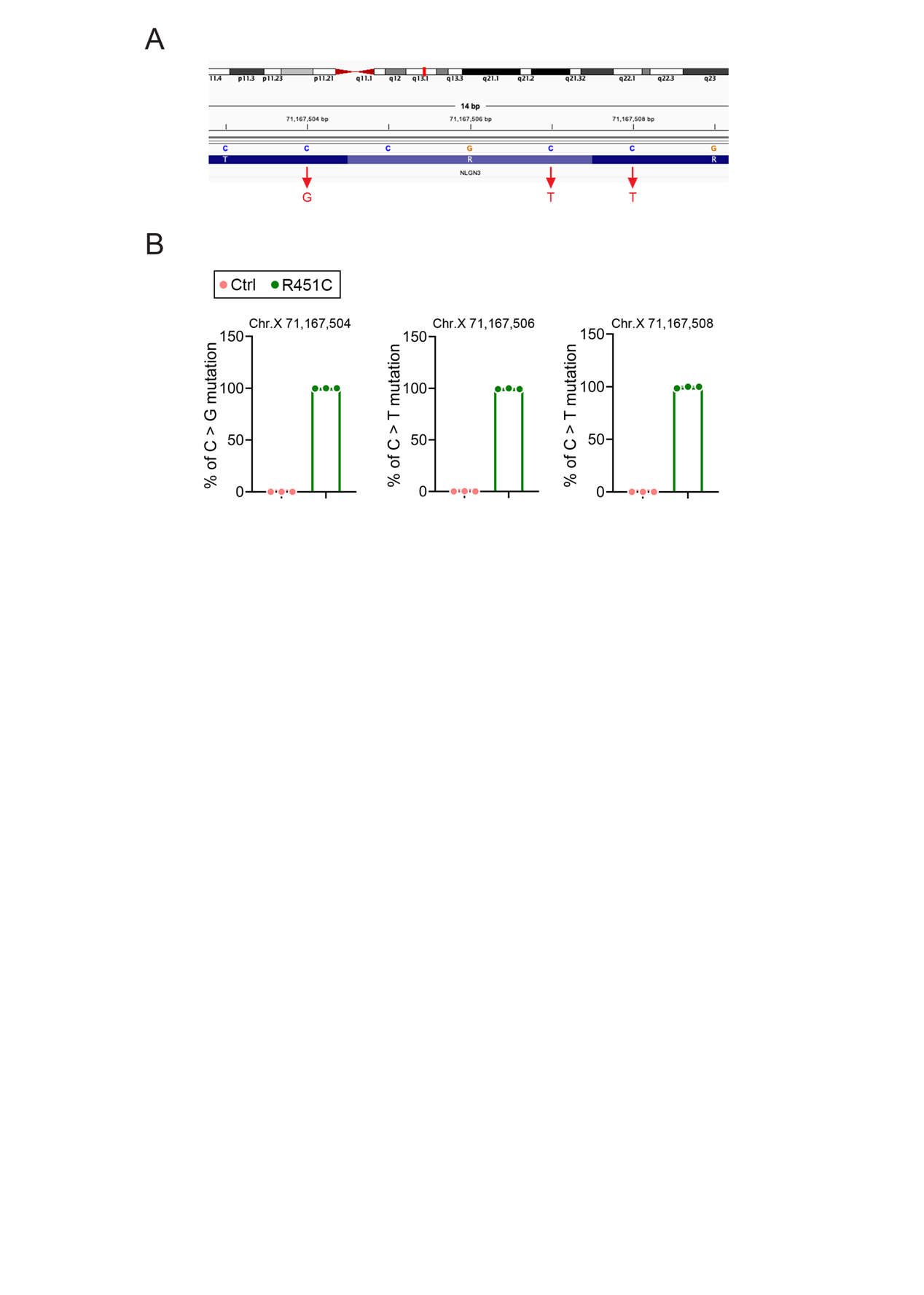


**Fig. S3** Characterization of NLGN3 R451C mutation in purified astroglia.

**A** Illustration of mutations in NLGN3 R451C cell line.

**B** Quantification of the percentage of mutations in total RNA for bulk RNA sequencing (n = 3 batches of organoids for both groups).
